# 6.Q. Skills building seminar: Integrating social inequalities in the burden of disease framework

**DOI:** 10.1093/eurpub/ckac129.399

**Published:** 2022-10-25

**Authors:** Organised by: COST Action CA18218– European Burden of Disease Network, Chair persons: Brecht Devleesschauwer (Belgium)

## Abstract

Driven by the influential Global Burden of Disease (GBD) study, the burden of disease (BOD) approach has gained wide interest at national and international level to quantify the state of health and health inequalities. Central to the BOD approach is the Disability-Adjusted Life Year (DALY) metric, which quantifies the health impact of diseases, injuries and risk factors as the number of healthy life years lost compared to a counterfactual scenario of perfect health all life long. The BOD approach offers a valuable platform to quantify social inequalities in health, i.e., differences in health status by socioeconomic and sociodemographic characteristics. This is highly relevant, as health inequalities penalising socially disadvantaged groups are one of the most consistent, and persistent, findings in epidemiology, for almost every health outcome and socioeconomic indicator. Monitoring social inequalities in health is therefore a key priority for national health authorities. There are different ways by which social inequalities can be integrated in the BOD framework, but all come with important data challenges. Individual-level stratification is a common approach for quantifying inequalities by age and sex, but is more challenging for other sociodemographic and socioeconomic indicators. Area-level stratification allows for ecological analyses between BOD estimates and indices of social deprivation. Social inequalities can also be assessed using comparative risk assessment, by which the relative risk for adverse health outcomes in function of social position is to be quantified. This skills building workshop will present the methods that have been applied in different national burden of disease studies to include social inequalities, including a discussion of their strengths and weaknesses. By providing a step-by-step presentation of how the methods have been applied, attendees will gain unique insights in the different ways by which social inequalities can be integrated in the BOD framework.

**Key messages:**

• The burden of disease framework offers a valuable platform to quantify and monitor social inequalities in health, which is a key priority for health authorities.

• Attendees will receive an overview of the different ways by which social inequalities can be integrated in the burden of disease framework, including a discussion of their strengths and weaknesses.

